# Distribution characteristics on droplet deposition of wind field vortex formed by multi-rotor UAV

**DOI:** 10.1371/journal.pone.0220024

**Published:** 2019-07-22

**Authors:** Shuang Guo, Jiyu Li, Weixiang Yao, Yilong Zhan, Yifan Li, Yeyin Shi

**Affiliations:** 1 College of Engineering, South China Agricultural University, Guangzhou, China; 2 National Center for International Collaboration Research on Precision Agricultural Aviation Pesticides Spraying Technology, Guangzhou, China; 3 Department of Biological Systems Engineering, University of Nebraska-Lincoln, Lincoln, Nebraska, United States of America; Washington University in Saint Louis, UNITED STATES

## Abstract

When the unmanned aerial vehicle (UAV) is used for aerial spraying, the downwash airflow generated by the UAV rotor will interact with the crop canopy and form a conical vortex shape in the crop plant. The size of the vortex will directly affect the outcome of the spraying operation. Six one-way spraying were performed by the UAV in a rice field with different but random flying altitude and velocities within the optimal operational range to form different vortex patterns. The spraying reagent was clear water, which was collected by water sensitive paper (WSP), and then the WSP was analyzed to study the droplets deposition effects in different vortex states. The results showed that the formation of the vortex significantly influenced the droplet deposition. To be specific, the droplet deposition amount in the obvious-vortex (OV) state was about 1.5 times of that in the small-scale (SV) vortex state, and 7 times of that in the non-vortex (NV) state. In the OV state, the droplets mainly deposited directly below and on both sides of the route. The deposition amount, coverage rate and droplet size increased from top to bottom of the crops with the deposition amount, coverage rate, and volume median diameter (VMD) ranging 0.204–0.470 μL/cm^2^, 3.31%-7.41%, and 306–367μm, respectively. In the SV state, droplets mainly deposited in the vortex area directly below the route. The deposition amount in the downwind direction was bigger than that in the upwind direction. The maximum of deposition amount, coverage rate and droplet size were found in the middle layer of the crops, the range are 0.177–0.334μL/cm^2^, 2.71%-5.30%, 295–370μm, respectively. In the NV state, the droplet mainly performed drifting motion, and the average droplet deposition amount in the downwind non-effective region was 29.4 times of that in the upwind non-effective region and 8.7 times of the effective vortex region directly below the route. The maximum of deposition amount, coverage rate and droplet size appeared in the upper layer of the crop, the range are 0.006–0.132μL/cm^2^, 0.17%-1.82%, 120–309μm, respectively, and almost no droplet deposited in the middle and lower part of the crop. The coefficient of variation (CV) of the droplet deposition amount was less than 40% in the state of obvious-vortex and small-scale vortex, and the worst penetration appeared in the non-vortex amounting to 65.97%. This work offers a basis for improving the spraying performance of UAV.

## 1 Introduction

Pests are prominent issues in crop production [1–2]. According to the data released by FAO, about 30% of the crop loss worldwide is caused by weeds, diseases and insect pests annually. Chemical spraying remains the most effective method for pest control [3]. Conventional land-spraying manners including manual backpack spraying and ground-based sprayer are either inefficient, labor intensive, hazardous for human health, or costly and limited by topography and canopy height [4–6]. Aerial applicators are widely used in conjunction with the ground-based sprayers in North America, Europe and some other areas for farms very large acreage; however, they are not feasible for small farms or orchards with complex terrain and field conditions. Unmanned aerial vehicle (UAV) based agricultural aviation technology finds its unique applications in those area [7–9]. Requiring no specific take-off and landing sites, rotary-wing UAVs, especially, can operate flexibly and effectively in complex terrains such as paddy fields, hills and mountains, and quickly respond to the outbreak of pests [10–11].

The effectiveness of droplet deposition is one of the most concerned issue in UAV spraying operation. In the process of spraying with UAV, the droplets permeate to the crop canopy and function. But droplets drift often occurs in practice, which wastes pesticides, reduces the control effect, and even causes pollution and poisoning [12]. The droplet deposition level on target crops is a critical index of evaluating spraying effects. Thus, increasing droplet deposition amount is important to the improvement of operations [13].

The droplet deposition is significantly influenced by the aerial nozzle. As a core component of the spraying system, the nozzle with good performance can effectively increase the droplet deposition [14]. Fritz et al. [15] tested the droplet deposition effects of three types of nozzles, and studied the effect of spraying speed and droplet size on deposition. However, a fixed-wing manned aircraft (AT-402B) was used in the test, which was significantly different from the rotor UAV. Kirk [16] analyzed the relationship between nozzle types, spray parameters and droplet drift, and established the atomization parameter model. But it was not an actual flight test but a simulation test carried out in a nozzle test device equipped with a laser spectrometer. Derksen et al. [17] conducted a spraying test in a soybean field to figure out the effect of nozzle types and spraying volume on droplet deposition. These researches concentrated on the influence of inherent physical parameters such as nozzle types and droplets, and neglected the impact of flight parameters on droplet deposition after the droplets were sprayed from the nozzle.

The flight parameters are also important that cannot be ignored, scholars have conducted researches on this. Zhang et al. [18] regulated the height and velocity of the WPH642 unmanned helicopter and studied the effect of spraying parameters on droplet deposition distribution on rice canopy in the rice spraying test, where deposition amount was not directly measured but was reflected through the temperature change before and after spraying by using infrared thermal imager. Chen et al. [19] used additive aqueous solution to conduct an experiment about the influence of spraying parameters on droplet deposition in the paddy field during a small unmanned helicopter operation. The result indicated that flight height and velocity remarkably influenced the average deposition amount of droplet. Considering that the flight parameters during the UAV operation are not constant, Wang et al. [20] tested the root mean square error and coefficient of variation of flight height and velocity by adopting four different UAVs and explored the influence of flight parameters on deposition distribution. These researches focused on the impact of flight parameters without studying the interaction mechanism between the UAV and the crop canopy.

Generated under the interaction of the high-speed rotation of the rotor and the air, the rotor wind field directly connects the UAV with the crop canopy, which influences the droplet deposition. Xue et al. [21] analyzed the difference of droplet deposition between the upper layer and lower layer of the rice plant, and explored the influence of the wind field on the penetrability of the droplet, but they hadn’t studied how the distribution and morphology of the wind field work. Chen et al. [22] investigated the influence of rotor wind field on the uniformity and penetration of droplet deposition in three spatial directions of X, Y and Z by using wind velocity sensor. The results showed that X and Y-direction wind field hardly influenced the droplet deposition while Z-direction wind field significantly did. But the overall distribution of the wind field still requires further study. Yang et al. [23] established a three-dimensional mathematical model of the downwash airflow when the multi-rotor UAV was hovering, then analyzed the motion characteristics of droplets, which provided guidance for related researches. These researches mainly explored how the rotor wind field influenced droplet deposition on crop canopy. The rotor wind field is the link between the UAV and the crop canopy, and vortex is the direct outcome of the wind field on the crop canopy.

The downwash airflow generated by the rotor provides the necessary lift for the UAV, and simultaneously, mixed with droplets, causes with the rice to fall around, which forms a conical vortex shape in the rice plant. Li et al. [24–26] elaborated on the feature of airflow during UAV performance and clarified that the vortex formed in rice and wheat plants was a typical feature of the low-altitude spraying of the rotor UAV. When the droplet size is within a certain range, the spatial mobility of the droplet is primarily determined by the distribution of the wind field [27–28]. Only when the wind field interacts with the crop canopy can the droplets deposit on the crop canopy, so the formation of the vortex is an important index of whether the wind field effectively interacts with the canopy. Some efforts have been made by the research community centering on the droplet deposition directly below the route of the UAV. However, the influence of vortex on droplet deposition and the droplet deposition inside and outside the vortex have not been covered, which are discussed in this article. Different vortex states indicate the level of the interaction between the wind field and the canopy. The relationship between the state of vortex and the droplet deposition amount is evaluated in our work.

This work is aimed to study the effect of vortex state on droplet deposition and different vortex state were formed by setting different flight height and velocities. A quadcopter UAV was used to spray reagent in the rice field. This study of vortex is of great significance to improve precise operation of UAV for plant protection.

## 2 Materials and methods

### 2.1 Experimental site

The experiment was conducted at the hybrid rice seed production base (18°53′52.65″N, 108°40′54.92″E) in the Bumo Village, Gancheng Town, Dongfang City, Hainan Province, China (the field is private land, and the owner of the land gave permission to conduct the study on this site). The test crop was hybrid rice Longliangyou 534 in the mature period with a planting density of 1.8×10^5^ plants per hm^2^ and planted in a narrow-and-broad-row pattern (140 and 60 cm alternately). The average planting height, plant spacing and row spacing were 100–115 cm, 25 cm and 15 cm, respectively.

### 2.2 Materials and devices

**Fig 1:**
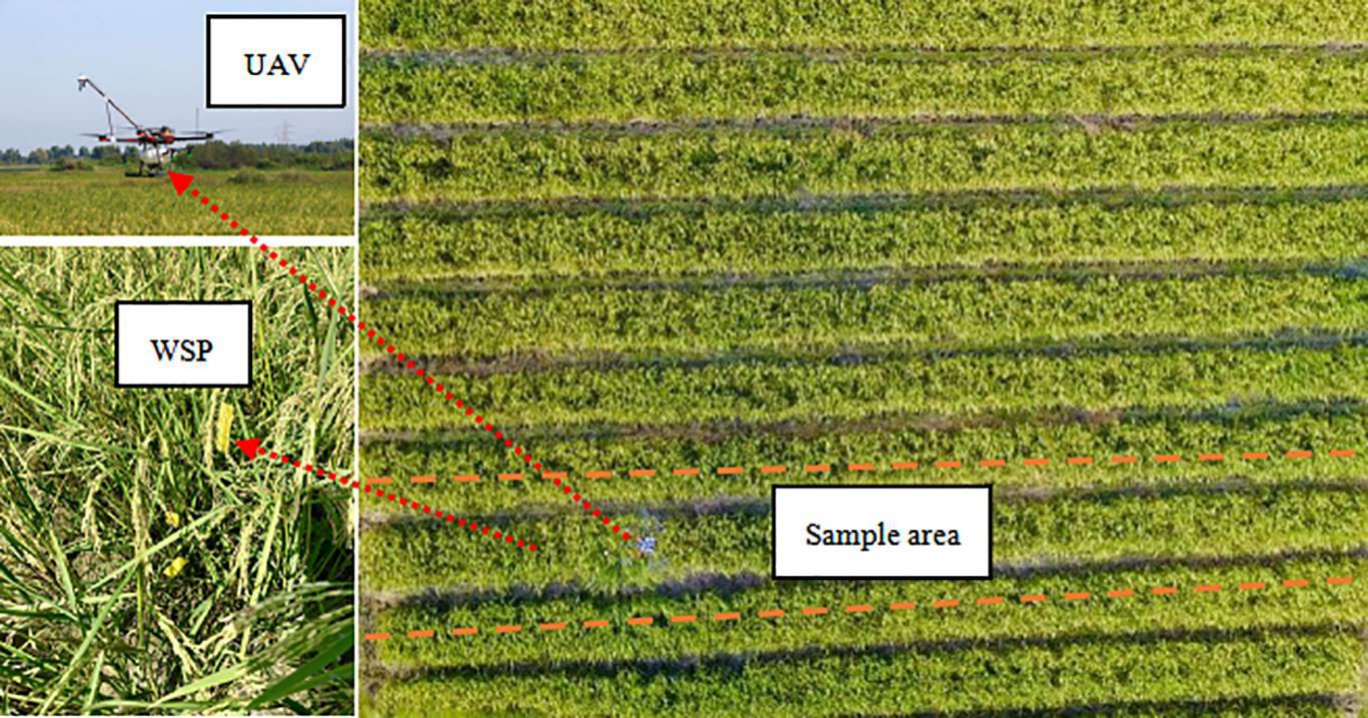
UAV and test site.

The UAV used in the experiment was a quadcopter electric UAV developed in the National Center for International Collaboration Research on Precision Agricultural Aviation Pesticides Spraying Technology of South China Agricultural University, Guangzhou, China. At the same time, a Phantom 4 Pro UAV (developed by Shenzhen DJI-Innovations Technology Co., Ltd, Shenzhen, China) was used to record the vortex formed under the influence of rotor wind field at the height of 20–25 meter above the field. The pesticide reagent was water, and the sample collection cards, sized 76×26 mm, were water sensitive paper (WSP) (produced by Syngenta Crop Protection LLC, Basel, Switzerland). The UAV and the spraying test sites are shown in Fig 1. A light airborne BeiDou RTK differential system capable of RTK differential positioning was integrated in the UAV system. Data were acquired every 0.1 second and actual operation trajectories were drawn based on the real-time flight parameters as references for spray effect analysis. The specifications of the UAV and airborne equipment are listed in Table 1. Four XR-110015 pressure flat fan-shaped nozzles (Spraying System Co., Illinois, USA) were symmetrically arranged vertically downward on both sides of the UAV at a spacing of 600 mm. The performance of the nozzles was tested before the field experiment, at a working pressure of 0.35 MPa, the DV0.5 of the four nozzles is 120, 124, 111, 117μm, respectively, the total measured flow rate of four nozzles marked 2.88 L/min.

**Table 1:** Specifications of the UAV and carrying equipment.

Main parameter	Norms and numerical
Type	Four-rotor electric UAV
Size/ mm×mm×mm	1000×1000×495
Rotor diameter/ mm	750
Maximum pesticide tank load/ L	10
Recommended flight height/ m	1~6
Recommended flight velocity/ (m·s^-1^)	0~8
Effective swath/ m	4~6
BeiDou plane accuracy/ mm	(10+5×D×10^−7^)^[a]^
BeiDou elevation accuracy/ mm	(20+1×D×10^−6^)^[a]^

Note

^[a]^ D in parentheses refers to actual distance measured by BeiDou, based on the unit of km.

### 2.3 Experimental method

**Fig 2:**
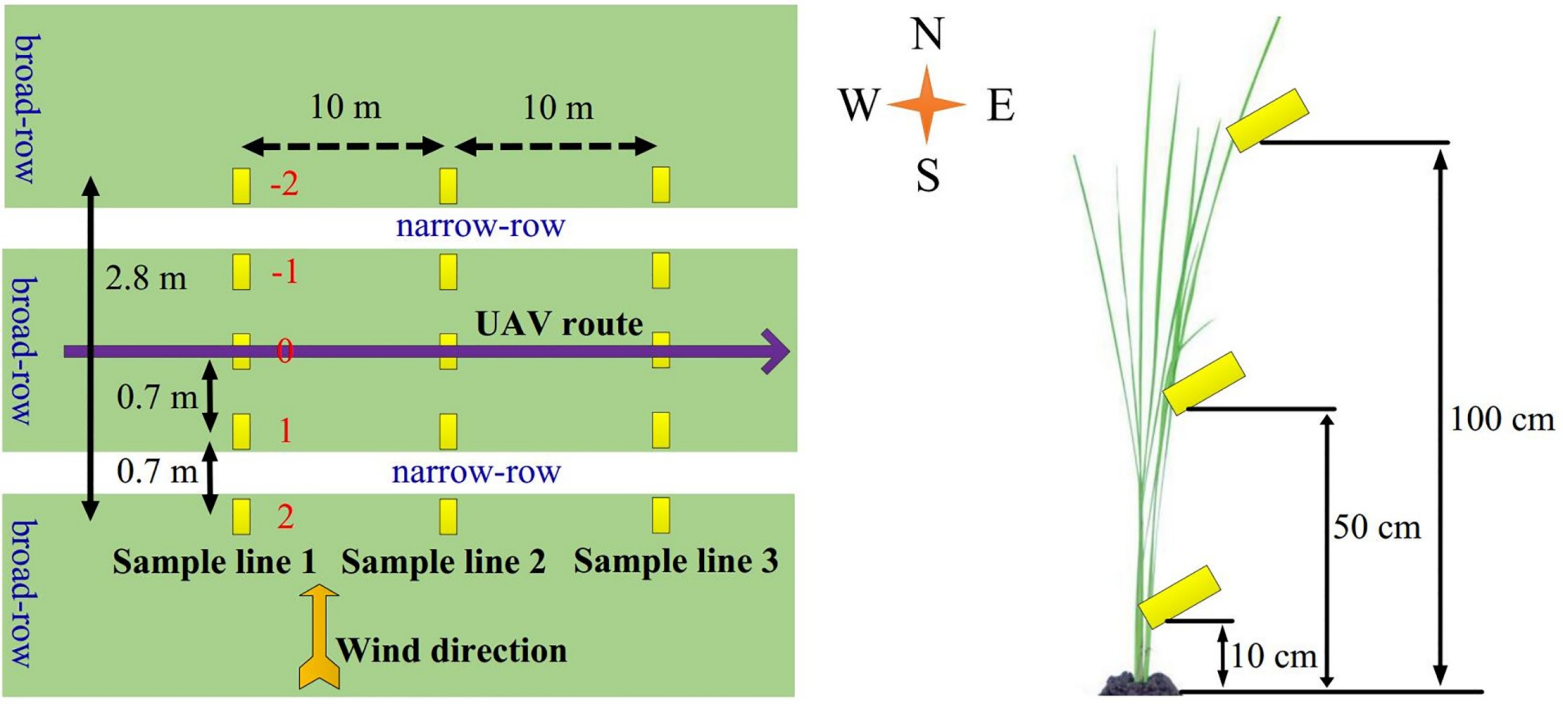
Schematic diagram of test.

The spray test was conducted in a 60×60 m unobstructed paddy field. As indicated in Fig 2, there were three 2.8-meter-long droplet collection lines with a spacing of 10 m. Sufficient take-off acceleration distance was reserved in front of the first collection line. Each line included five sampling points, numbered as -2, -1, 0, 1 and 2 from left to right. The region covered between sampling points -2 and -1 was the upwind non-effective vortex region; the region covered between sampling points -1 and 1 was the effective vortex region in which sampling point 0 was on the UAV flight route; the region covered between sampling points 1 and 2 was the downwind non-effective vortex region. Three pieces of WSP were placed at each sampling point at the height of 10 cm, 50 cm and 100 cm above the ground, respectively. All WSPs were placed facing the windward direction. Two Kestrel 5500 Link micro weather stations (Nielsen-Kellerman Co., Boothwyn, USA) were used in the experiment to record the real-time temperature, humidity, wind velocity, wind direction and other meteorological information during the experiment every 2 seconds. One station was placed at the bottom of the canopy, the other was placed at the field and 2 m above the ground. Six one-way spraying (1#~6#) were performed by the UAV from west to east with different but random flying altitude and velocities within the optimal operational range to form different vortex patterns.

### 2.4 WSP collection and spraying effectiveness evaluation

After every spraying test, an operator wearing disposable gloves immediately collected the WSPs in turns after the droplets became dry, placed them in marked envelopes, stored in a cool condition and transferred them to the laboratory for further study. WSPs were analyzed by using image processing software Deposit Scan (developed by USDA, Wooster, USA). The deposition, D_V0.5_ and deposition density were studied. Meanwhile, mean deposition, mean deposition density and coefficient of variation (CV) were also calculated. The D_V0.5_, usually known as the volume median diameter (VMD), refers to the median for the droplet size when the accumulation of all droplets from small to large size is equivalent to 50% of the total volume of the droplets. It is a critical index to measure the size of the droplet [29].

The CV was calculated to study the distribution uniformity of droplet deposition among sampling points. The smaller the CV is, the better the uniformity of droplet deposition is. It was calculated by the following equation:

$$CV=\frac{S}{\bar{X}}\times 100\%$$ (1)

$$S=\sqrt{\sum_{i=1}^{n}{\left(X_{i}-\bar{X}\right)}^{2}/(n-1)}$$ (2)

S represents the standard deviation. *X*_*i*_ refers to the deposition value of every sampling point (μL/cm^2^). $\bar{X}$ stands for the average deposition value of every sampling point (μL/cm^2^), n is the number of sampling points.

## 3 Results and discussion

### 3.1 Meteorological data

The light airborne BeiDou RTK difference system accurately recorded the time of each flight test while the micro weather stations recorded the meteorological data every 2 seconds, which are shown in Table 2. The ambient temperature, humidity and wind velocity were stable during the test. The average temperature and humidity were 27.0°C and 68.7%, while the wind velocity remained in the range of 2.3–2.8 m/s. Although there were some changes in the wind direction, the deviation range was within ±30°, which was in line with the NY/T 3213–2018 test standard [30]. In addition, it can also be found that the temperature measured by the micro weather stations in the lower part of the canopy was slightly higher than that of the field, and the wind velocity was slightly lower than that of the field due to the transpiration of crops and the mutual occlusion between plants. As time went on, the ambient humidity gradually increased during the operation, and the humidity in the lower part of the canopy increased obviously.

**Table 2:** Summary of meteorological data for each test.

Test	Operation time	Meteorological acquisition location	Temperature(°C)	Humidity (%)	Wind velocity (m/s)	Wind description	Wind Angle Deviation^[a]^ (°)
1#	12:02–12:03	Lower canopy	29.7	61.3	1.72	NE	22.7
		Field	27.3	67.1	2.18		
2#	14:15–14:16	Lower canopy	29.8	64.1	1.22	N	5.1
		Field	28.1	66.1	2.32		
3#	14:55–14:56	Lower canopy	28.6	65.7	0.45	NE	17.3
		Field	27.9	66.6	2.82		
4#	15:25–15:26	Lower canopy	27.5	69.3	0.30	NE	15.6
		Field	27.1	68.5	2.29		
5#	15:53–15:54	Lower canopy	26.1	71.2	0.36	NE	21.6
		Field	26.4	69.6	2.52		
6#	16:27–16:28	Lower canopy	24.1	77.8	0.33	N	9.5
		Field	25.3	74.1	2.74		

Note

^[a]^Wind angle deviation corresponds to angle of wind relative to the droplet sampling line (°).

### 3.2 The result of vortex state determination

**Fig 3:**
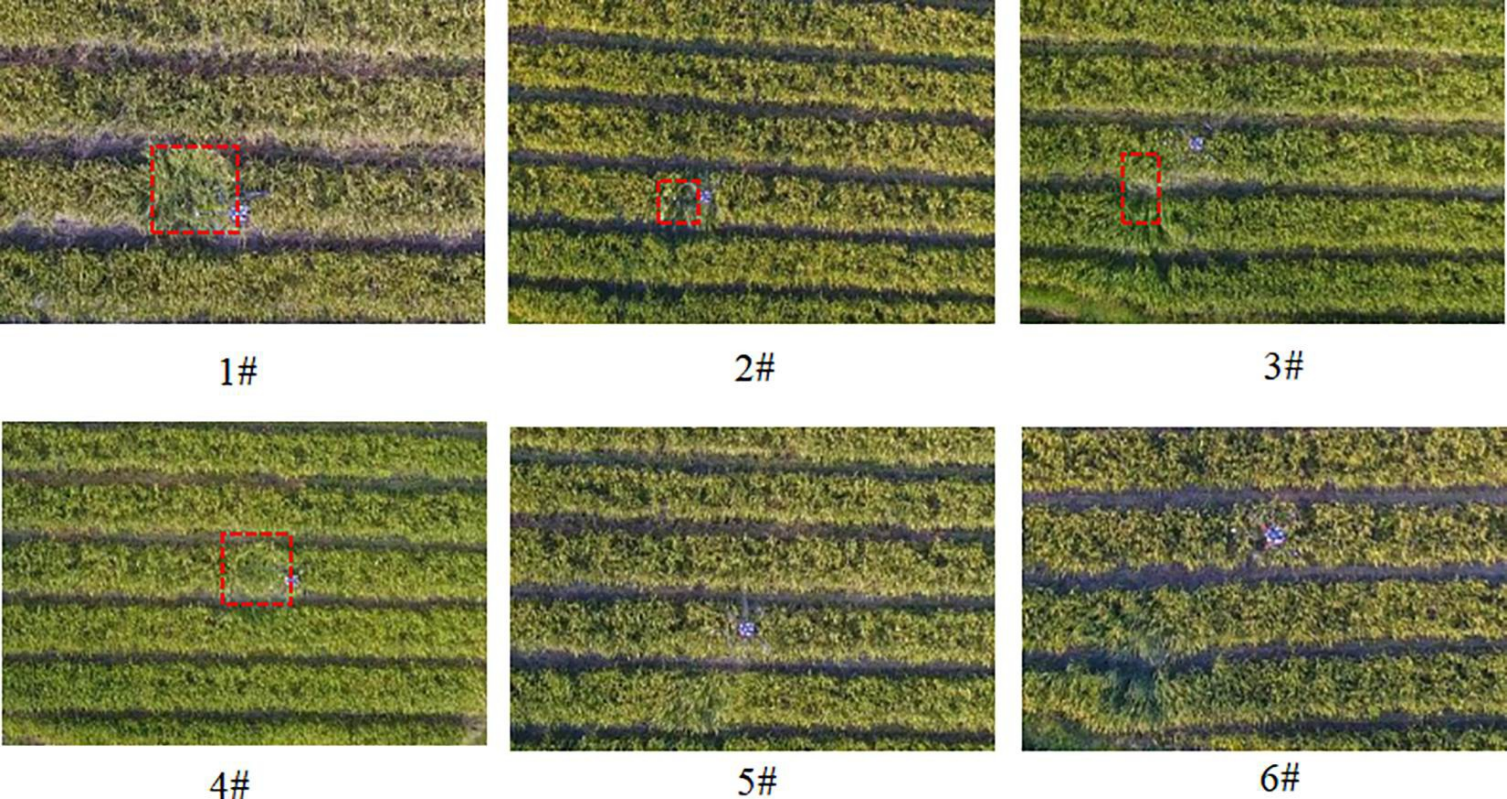
Aerial photographs of vortex-state of each test.

The video images of each vortex state obtained by the Phantom 4 Pro UAV are shown in Fig 3. The red dotted area is the vortex area formed by the influence of UAV rotor flow field. The longest side length of the red dotted area is recorded as D, and the maximum rotor wheelbase size of the drone is recorded as L (L = 1.75 m). The vortex state is defined by the value of D/L. When the value of D/L is greater than 1, the vortex state is recorded as an obvious-vortex (OV) state, when the value of is between 0 and 1, the vortex state is marked as a small-scale vortex (SV) state, and if the value of is 0, the vortex state is marked as non-vortex (NV) state. According the video images, the vortex state and size were identified and compared to each other. The vortex states of different tests were ordered from large to small as follows: 1#, 4#, 3#, 2#, 5#, and 6#. 1# and 4# were OV state, 3# and 2# were SV state, and 5# and 6# were NV state.

The light airborne BeiDou RTK differential system accurately recorded the height and velocity parameters of each flight test, which were shown in Table 3. The results indicated that the parameters were within the range of normal flight operation and different vortex states were created as expected. The average flight height was 2.85 meter, with the minimum height of 1.51 meter (2#) and the maximum height of 4.90 meter (6#). The flight velocity was kept in the range of 1.11–3.43 m/s, and was almost the same when flying over three droplet collection lines in one test. As the experiment required, the effective application distance of each spraying operation was longer than 30 m.

**Table 3:** Summary of vortexes and corresponding flight parameters for various test sorties.

Vortex state	Test	Mean flight height^[a]^ (m)	CV of flight height (%)	Mean flight velocity (m/s)	CV of flight velocity (%)	Effective application distance (m)
OV	1#	1.28	16.55	1.30	10.46	32.66
	4#	2.67	10.33	2.34	5.88	34.70
SV	3#	2.52	8.45	1.68	17.32	33.24
	2#	1.51	25.11	2.98	8.64	31.20
NV	5#	4.23	18.10	1.11	19.02	35.68
	6#	4.90	6.79	3.43	10.05	33.21

Note

^[a]^Mean flight height corresponds to the distance of UAV nozzle relative to the top of the crop canopy (m).

### 3.3 Analysis of droplet deposition

#### 3.3.1 Overall droplet deposition effect of each sort

**Fig 4:**
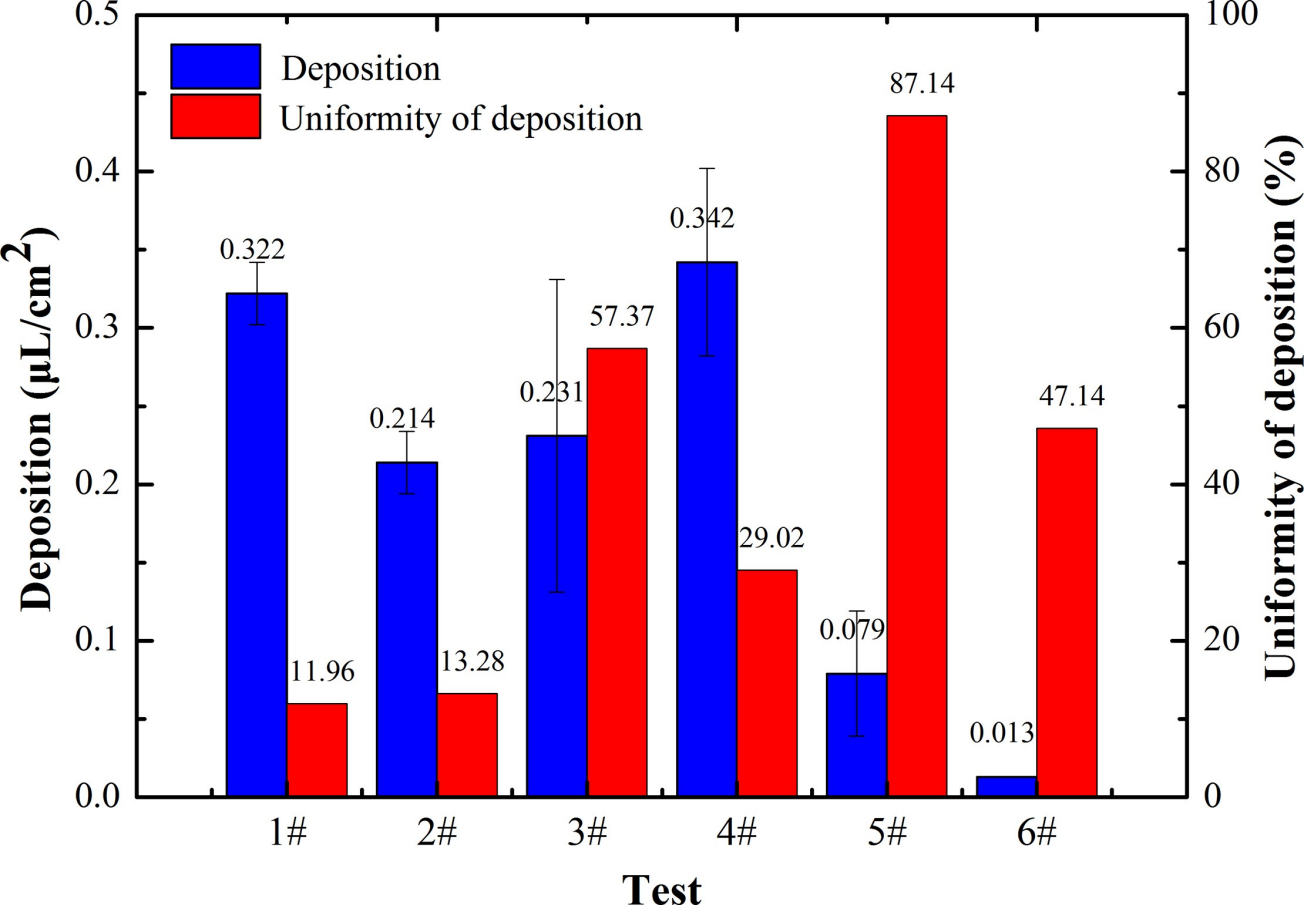
Overall effect of droplet deposition in each test.

The average droplet deposition and uniformity of each spraying operation are shown in Fig 4. The droplet deposition is an important indicator that reflects the actual deposition level of the sprayed droplets. The average droplet deposition of each test obtained by WSP analysis was sequenced from large to small as follows: 4# (0.342 μL/cm^2^), 1# (0.322 μL/cm^2^), 3# (0.253 μL/cm^2^), 2# (0.214 μL/cm^2^), 5# (0.079 μL/cm^2^), and 6# (0.013 μL/cm^2^). 4# and 1# were OV state, 3# and 2# formed SV state, and 5# and 6# were NV state. Generally speaking, when the spraying operation is performed at a lower flight height (2#) or a slower flight velocity (3#), the droplet deposition level is higher. However, we found that the detected deposition amount of 2 # and 3 # was not the biggest with NV state, while that in 1 # and 4 # was the biggest with OV state, which indicated vortex state influences droplet deposition. It shows that deposition quality in the OV state was better than that in the SV state, and that in the NV state was the worst. In other words, the formation of an stronger vortex in the effective spraying area facilitated droplet deposition.

The deposition uniformity of droplets is another typical index to evaluate the deposition effect. The smaller the CV is, the more well-distributed the droplets are. 1# (OV state), 2# (SV state) and 4# (OV state) ranked the first with the CV of deposition uniformity less than 30%, 6# (NV state) and 3# (SV state) came second, and 5# (NV state) was the worst with the CV of deposition uniformity as high as 87.14%. The results further reveal that the more obvious the vortex state was, the more well-distributed the droplets were.

#### 3.3.2 Distribution characteristics of droplet deposition along the depth dimension

Since the WSPs were placed 100 cm, 50 cm and 10 cm above the ground, the sampling locations were divided into upper (UL), middle (ML) and lower layers (LL). Table 4 shows characteristics of droplet deposition for each layer through the analysis of deposition amount, coverage per unit area and VMD.

**Table 4:** Characteristics of droplet deposition for each layer of each test.

Test	Sampling location	Mean deposition(*μ*L/cm^2^)	Distribution uniformity (%)	Mean coverage(%)	Distribution uniformity (%)	Mean VMD(*μ*m)	Distribution uniformity (%)
1#	UL	0.204±0.07 b	58.14	3.31±1.12 b	58.63	306±20.43 a	11.55
	ML	0.319±0.01 ab	6.68	5.60±0.23 ab	7.25	317±8.52 a	4.65
	LL	0.444±0.09 a	36.24	7.41±1.32 a	30.89	345±44.74 a	22.46
2#	UL	0.216±0.03 a	24.66	3.75±0.58 a	26.74	298±28.97 a	16.84
	ML	0.243±0.03 a	23.95	4.30±0.45 a	18.22	317±0.58 a	0.32
	LL	0.181±0.06 a	58.16	3.12±0.96 a	53.30	295±5.75 a	3.38
3#	UL	0.180±0.06 a	56.59	2.71±0.78 a	50.13	318±4.55 a	2.47
	ML	0.334±0.19 a	98.90	5.30±2.85 a	93.11	370±49.89 a	23.38
	LL	0.177±0.03 a	25.39	2.94±0.31 a	18.12	370±10.25 a	5.88
4#	UL	0.284±0.06 a	36.44	4.63±0.82 a	30.82	315±24.61 a	13.52
	ML	0.273±0.05 a	32.31	4.64±0.78 a	29.09	317±5.96 a	3.25
	LL	0.470±0.16 a	59.97	6.64±1.70 a	44.41	367±24.37 a	11.49
5#	UL	0.132±0.06 a	81.36	1.82±0.78 a	73.69	309±6.74 a	3.78
	ML	0.065±0.03 a	88.28	0.92±0.43 a	80.49	277±34.41 ab	21.55
	LL	0.041±0.03 a	120.07	0.63±0.36 a	99.61	219±25.14 b	19.91
6#	UL	0.022±0.01 a	68.10	0.49±0.15 a	53.02	144±12.86 a	15.45
	ML	0.010±0.00 a	52.84	0.24±0.08 a	62.19	141±9.72 a	11.95
	LL	0.006±0.00 a	34.89	0.17±0.02 a	22.71	120±17.48 a	25.14

Note: The data in the table are mean±SE. Columns with the same letter are not significantly different (p<0.05).

The droplet deposition in 1# and 4# applications increased along the depth dimension with the heaviest depositions at the LL (0.444 μL/cm^2^ and 0.470 μL/cm^2^ for the two applications, respectively). The droplet depositions in the 2# and 3# applications was lightest in the LL which was 0.181 μL/cm^2^ and 0.177 μL/cm^2^, respectively, and heaviest in the ML which was 0.243 μL/cm^2^ and 0.334 μL/cm^2^, respectively. 5# and 6# had a small amount of deposition in the UL and even lighter amount in the ML and the LL. The strongly interaction between the wind field and the plants in 1# and 4# formed OV in the rice plants, the canopy blown open better exposed the stems in the wind and helped a better delivery of the droplets to the canopy bottom. Compared to 1# and 4#, the weak interaction between the wind field and the plants in 2# and 3# formed SV, where the canopy was not completely blown open, so most of the droplets deposited in the ML while few reached the LL. 5# and the 6# formed no vortex because the rotor wind field did not interact with the canopy at all, so there was only a small amount of droplet deposition in the UL of the canopy. Moreover, there was a significant difference of deposition amount and the coverage per unit area in the UL and the LL in 1#, indicating that the droplets effectively reached the LL of the plant, and the stronger vortex state influenced droplet deposition.

The coverage per unit area in1# and 4# increased from the UL to the LL, and the coverage per unit area in the LL was 7.41% and 6.64% respectively. The coverage per unit area in 2# and 3# reached the maximum in the ML, which was 4.30% and 5.30%. The coverage per unit area in 5# and 6# in the UL was 1.82% and 0.49%, respectively, while that in the ML and the LL was small. The coverage per unit shared the same distribution characteristics with deposition amount, which further showed that the more obvious the vortex state was, the higher the coverage per unit area was, and that the vortex state influenced the coverage of the droplet per unit area.

The uniformity of deposition and coverage per unit area were found best in the ML in 1# (OV state), marking 6.68% and 7.25%. In 5# (NV state), the deposition uniformity was the worst in the LL with the CV of deposition amount reaching 120.07% and CV of per unit area coverage reaching 99.61%. As it is shown in Section 3.3.1, the deposition uniformity in 1# and 4# was significantly better than that in 5# and 6#, which indicated that the stronger vortex state contributes to the uniform deposition of droplets in various parts of rice plants.

The droplet size shared a similar distribution characteristics with the coverage per unit area and deposition amount. The VMD in 1# fell into the range of 306–345 μm, increased from the UL to the LL, and reached the maximum in the LL. That is because the crop canopy was completely blown off, the LL of the crop was exposed in the OV state, and the droplets would quickly settle to the crop leaves under the action of a strong rotor wind field. The WSP in LL received more droplets than UL and ML, which was easy to cause droplets overlapping on the WSP surface. However, the existing droplets image processing technology is difficult to realize droplets segmentation and overlapping droplets recognition, so that the measured droplets size value is larger. Each layer in 4# and 1# had the same distribution trend, with the minimum VMD of 315 μm, and the maximum of 367μm. The VMD in 2# and 3# reached the maximum in the ML, marking 317 μm and 370 μm, respectively. The VMD in the UL in 5# and 6# were 309 μm and 144μm, respectively, and the VMD in the ML and the LL decreased from up to down. The minimum VMD in 5# was 219 μm, and that in 6# was 144 μm.

The minimum CV was that in the ML in 2#, marking 0.32%, which indicated that the droplet size distribution in this layer was the most uniform. The UL in 3#, the ML in 4#, the LL in 2#, and the UL in 5# showed small CV and uniform distribution. The distribution uniform in the ML and LL in 5#, ML in 3# and LL in 6# was poor, and the VMD’s distribution uniformity in the LL in 6# was the worst, marking 25.14%.

Evaporation is also an important factor affecting the deposition effect, but the effect is limited in this experiment. That is due to the droplet detection method adopted is WSP color rendering method. Once the droplet falls on the WSP, they will immediately develop color. This color development time is extremely short, and evaporation mainly occurs after the droplets have settled on the leaves. It can be considered that except for a small part of the droplets that would evaporate during the spraying, the droplets fell on the plants all can be detected by WSP before evaporation. This avoids the possibility of evaporation interference test results, so that accurate droplet deposition effect can be obtained.

#### 3.3.3 Distribution of droplet deposition across the flight path

**Fig 5:**
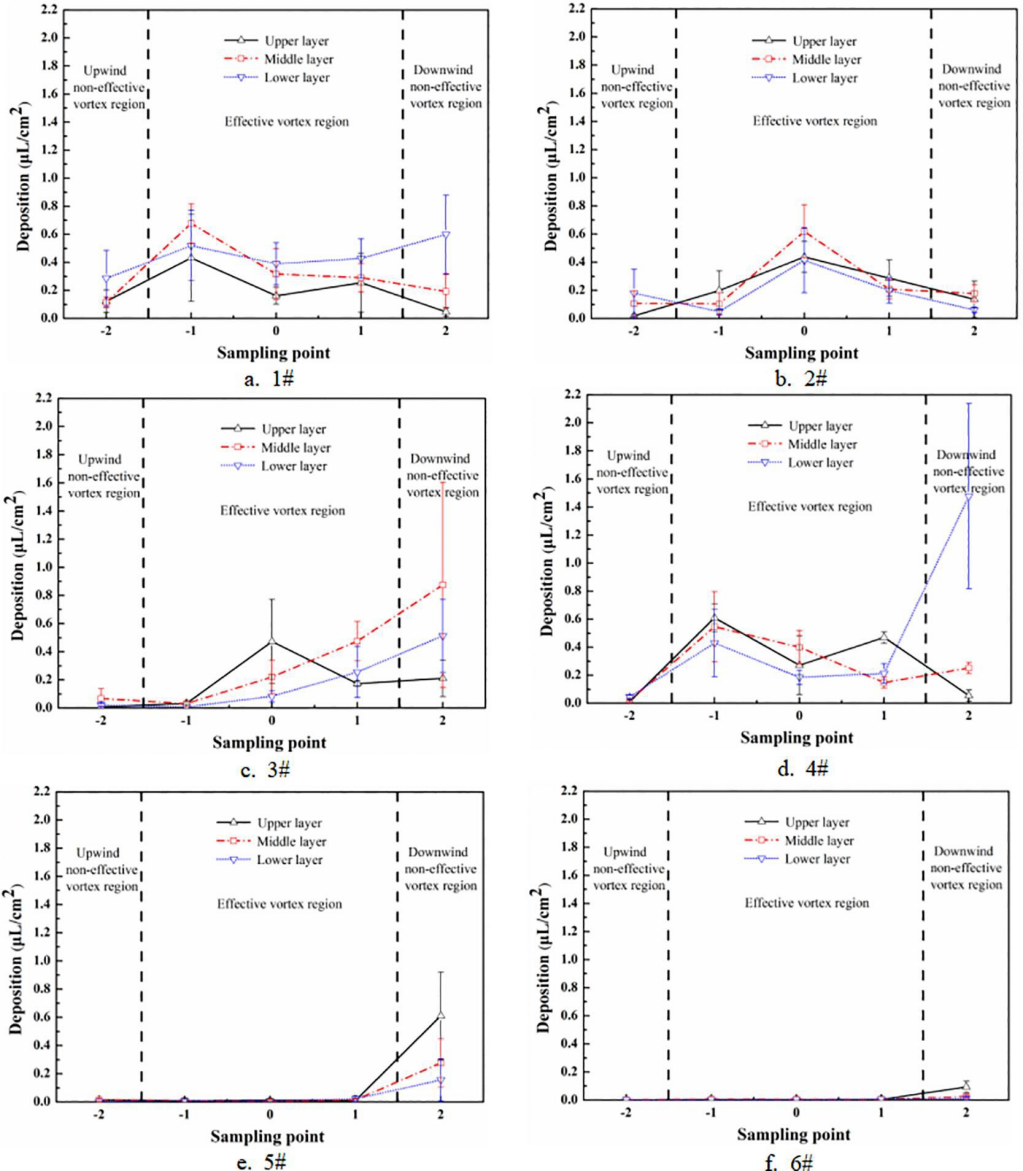
Characteristics of droplet deposition for each sampling point of each test.

According to the effective range of the vortex and wind direction, the sampling area was divided into three zones: the upwind non-effective vortex region, the effective vortex region and the downwind non-effective vortex region. Fig 5 presents the droplet deposition characteristics of each layer in every zone.

The x axis in Fig 5 represents the position of each sampling point, and the y axis refers to the droplet deposition amount in uL/cm^2^. It can be seen from the figure that the droplets mainly deposited in the effective vortex area and the downwind non-effective vortex region, which was as expected since the droplets drifted with the wind.

It can be seen from Section 3.2 that 1# and 4# were in the OV state. Fig 5A and 5D showed that the droplet deposition peaked on both sides in the effective vortex region, which meant the droplets mainly deposited below the aircraft route. A large amount of droplets deposited at sampling point -1 and 1 because a wide-range vortex covering the two sampling points was formed when the UAV blew open the rice plants directly below it. This indicated that the droplets were mainly deposited within the vortex or the desired region. In addition, since the exposed area of the LL of the plant was increased in the OV state, as Fig 5 shows, the deposition amount of each sampling points in the LL in 1# and 4# significantly increased with the wind direction, gradually approached or even exceeded that in the ML and the UL, and reached the maximum in the downwind non-effective vortex region, marking 0.599 μL/cm2 (1#) and 1.478 μL/cm2 (4#). The narrow-and-broad-row pattern (140 cm+60 cm) accounted for the results, it was learned from Figs 1 and 2 that there was a broad-row of 60 cm between the effective vortex region and the downwind non-effective vortex region. In the OV state, with the increase of exposed area in the LL in downwind non-effective vortex region, the droplet deposition amount increased simultaneously.

SV state was formed in 2# and 3#. As Fig 5B shows, every layer’s droplet deposition in 2# in the effective vortex region presented a single peak, which means that and the deposition amount peaked in the central line and decreased on both sides because the vortex functioned directly below the route. It is found from Fig 5C that only the deposition amount in the UL in 3# exhibited a single peak trend, while the deposition amount in the ML and the LL increased significantly along the wind direction. The flight height in 3# (2.52 m) was higher than that in 2# (1.51 m), so the droplets travelled longer in the air with reducing velocity and were easier to be influenced by the wind. Furthermore, compared with the OV state, in the SV state less rotor airflow directly interacted with the target canopy (the canopy directly below the UAV body), but easily drifted to the non-effective vortex region. In addition, the wind field exerted greater influence on the downwind region because the vortex volume in 3# was slightly more obvious than that in 2#, while the interaction between the wind field and the canopy in 3# was stronger than that in 2#, which attributed to the increase of deposition amount in the ML and the LL in the downwind non-effective vortex region.

No vortex was formed on the crop canopy below the UAV route in 5# and 6#. As Fig 5E and 5F presents, the WSP collected few droplets in the effective vortex because the droplets drifted with the wind to the downwind non-effective vortex region. According to the statistical calculation, the average deposition amount of one sampling point in the downwind non-effective vortex region was 29.4 and 8.7 times of that in the upwind non-effective vortex region and the effective vortex region respectively, which indicated a significant drift in 5# and 6#. Table 5 shows the results of the droplet penetrability of each test.

**Table 5:** Droplet penetrability of each test.

Test	1#			4#			3#			2#			5#			6#		
Vortex	OV						SV						NV					
Canopy layer	UL	ML	LL	UL	ML	LL	UL	ML	LL	UL	ML	LL	UL	ML	LL	UL	ML	LL
Mean deposition(μL/cm^2^)	0.204	0.319	0.444	0.284	0.273	0.470	0.180	0.334	0.177	0.216	0.243	0.181	0.132	0.065	0.041	0.022	0.010	0.006
Standard deviation	0.12			0.11			0.09			0.03			0.05			0.01		
Droplet penetrability (%)	37.35			32.41			39.01			14.61			59.85			65.97		

The CV along the depth dimension of the six flights is ordered from small to large as follows: 2# (SV state), 4# (OV state), 1# (OV state), 3# (SV state), 5# (NV state), and 6# (NV state). The smaller the CV is, the better the droplet distribution uniformity is. The CV in 1#, 2#, 3# and 4# was less than 40%, indicating sound permeability of the droplets. Of six tests, 5# and 6# without vortex demonstrated the poorest penetrability and the maximum of CV was found in 6# (65.97%). It is found that the stronger the vortex state is, the better penetrability in the vertical space is for better application effect.

## 4 Future work

The distribution of VMD in each layer is mentioned in section 3.3.2. Droplet size is one of the important indicators for evaluating the deposition effect. Li et al. [31] studied the influence of droplet size on the deposition and permeability on rice canopy, but did not take the wind field into account. In this paper we mainly explored the distribution characteristics of deposition amount in different vortex state. The relationship between the vortex state and the droplet size distribution characteristics needs to be further investigated. Only when the droplets sizes distribute within the optimal range can the best control effect be reached. In the future, efforts can be made to study the correlation of vortex state and droplet size distribution by determining the droplet size in various vortex states formed under the interaction of the rotor wind field and the canopy.

Meanwhile, according to the value of D/L, the vortex state is only divided into three ranges in this paper, OV (D/L>1), SV (0<D/L<1) and NV (D/L = 0). the range is broad, and there is a lack of correlation with other parameters. Therefore, in the following research, we will comprehensively consider the introduction of a variety of correlation parameters, and further refine the vortex state range, in order to obtain a more accurate vortex state suitable for UAV spraying operations. Researches are bound to provide guidance and theoretical basis for the spraying operation of UAV.

## 5 Conclusion

In this paper, a quadcopter UAV was used to form different vortex states by randomly setting six groups of flight height and velocity parameters. Six vortex states were formed according to the degree of interaction between the rotor wind field and the crop canopy. The droplet deposition amount and the distribution characteristics in each vortex state were analyzed. The conclusion from the experiment can be summarized as follows:

1. The flight parameters are not the decisive factors affecting the deposition, while the formation of vortex is more important. A suitable vortex state is more helpful to improve the quality of the operation. The average deposition amount in the OV (0.332 μL/cm^2^) was 1.5 times of that in the SV (0.234 μL/cm^2^) and 7 times of that in the NV (0.046 μL/cm^2^). The deposition amount with the stronger vortex was notably higher than that in non-vortex state.
2. The vortex size also influenced the droplet deposition. The stronger the vortex, the more the deposition, the higher the coverage per unit area and the better the deposition uniformity. In the OV state, the deposition amount (ranging 0.204–0.470 μL/cm^2^), the coverage (ranging 3.31%-7.41%) and the droplet size (VMD ranging 306–367μm) increased from top to bottom. In the SV state, the maximum of deposition (0.334 μL/cm^2^), coverage (5.30%) and VMD (370 μm) occurred in the ML of the crop, and the range of deposition, coverage and VMD are 0.177–0.334μL/cm^2^, 2.71%-5.30%, 295–370μm, respectively. In the NV state, the maximum of deposition (0.132 μL/cm^2^), coverage (1.82%) and VMD (309 μm) appeared in the UL while few droplets deposited in the ML and the LL, the range of deposition, coverage and VMD are 0.006–0.132μL/cm^2^, 0.17%-1.82%, 120–309μm, respectively. Moreover, the minimum distribution uniformity of deposition, coverage and VMD appeared in OV tests, marking 6.68%, 7.25% and 0.32%, respectively, while the maximum value appeared in the NV state, marking 120.07%, 99.61% and 25.14%.
3. In the OV state, the droplets mainly deposited in the vortex area directly below and on both sides of the route. In the SV state, droplets mainly deposited in the vortex area directly below the route, and the deposition amount in the downwind direction was higher than that in the upwind direction under the influence of the wind direction. The droplets mainly drifted away in the NV state, where the average deposition amount in the downwind non-effective vortex region was 29.4 times of that in the upwind non-effective vortex region and 8.7 times of that in the effective vortex region directly below the route.
4. Stronger vortex facilitated the droplet penetration in the depth dimension of the canopy. The CV of the deposition amount was less than 40% in the state of OV and SV, and the worst penetration appeared in the NV state, up to 65.97%. The more obvious the vortex state is, the better penetrability of the droplets in the vertical space for better application effect.
